# Sensing-Based Dynamic Spectrum Sharing in Integrated Wireless Sensor and Cognitive Satellite Terrestrial Networks

**DOI:** 10.3390/s19235290

**Published:** 2019-12-01

**Authors:** Jing Hu, Guangxia Li, Dongming Bian, Jingyu Tang, Shengchao Shi

**Affiliations:** 1 College of Communications Engineering, People Liberation Army Engineering University, No. 2 Biaoying, Qinhuai District, Nanjing 210007, China; jinghu_321@163.com (J.H.); Satlab13905177686@163.com (G.L.); bian_dm@163.com (D.B.); Ryan_TJY@163.com (J.T.); 2 Beijing Institute of Information Technology, Beijing 100094, China

**Keywords:** cognitive satellite, wireless sensor network, ergodic capacity, fading channel, opportunistic spectrum access, spectrum sharing

## Abstract

This paper presents a cognitive satellite communication based wireless sensor network, which combines the wireless sensor network and the cognitive satellite terrestrial network. To address the conflict between the continuously increasing demand and the spectrum scarcity in the space network, the cognitive satellite terrestrial network becomes a promising candidate for future hybrid wireless networks. With the higher transmit capacity demand in satellite networks, explicit concerns on efficient resource allocation in the cognitive network have gained more attention. In this background, we propose a sensing-based dynamic spectrum sharing scheme for the cognitive satellite user, which is able to maximize the ergodic capacity of the satellite user with the interference of the primary terrestrial user below an acceptable average level. Firstly, the cognitive satellite user monitors the channel allocated to the terrestrial user through the wireless sensor network; then, it adjusts the transmit power based on the sensing results. If a terrestrial user is busy, the satellite user can access the channel with constrained power to avoid deteriorating the communication quality of the terrestrial user. Otherwise, if the terrestrial user is idle, the satellite user allocates the transmit power based on its benefit to enhance the capacity. Since the sensing-based dynamic spectrum sharing optimization problem can be modified into a nonlinear fraction programming problem in perfect/imperfect sensing conditions, respectively, we solve them by the Lagrange duality method. Computer simulations have shown that, compared with the opportunistic spectrum access, the proposed method can increase the channel capacity more than 20% for $P_{av}=10\;\mathrm{dB}$ in a perfect sensing scenario. In an imperfect sensing scenario, $P_{av}=15$ dB and $Q_{av}=5$ dB, the optimal sensing time achieving the highest ergodic capacity is about 2.34 ms when the frame duration is 10 ms.

## 1. Introduction

Wireless sensor networks (WSNs) have been widely used in many fields, such as agriculture, monitoring, and geographical routing [1,2,3,4]. The nodes of wireless sensor networks can share the effective information with each other, and transmit the information to the remote monitoring hosts. For the obvious superiority in high data rate services and providing coverage in the remote areas with various users, satellite-based sensor network systems have gradually drawn the attention of researchers and become a new direction of sensor networks  [5,6,7,8].

Meanwhile, a fundamental challenge for satellite communications (SatCom) is the spectrum exploitation for the continuous growth of broadband applications and multimedia services [9,10,11,12]. To improve the spectrum efficiency, cognitive radio (CR) has exploited the spectrum sharing in terrestrial networks for more than ten years as a mainly approach, which is also a very promising candidate in SatCo [13,14,15]. In such a network, CR can be used not only in satellite networks, but also in the integrated satellite-terrestrial networks.

In CR networks, the legitimate licensed users are the primary users (PUs), and the unlicensed users are referred to as secondary users (SUs).  Generally, the approaches of CR are to permit the SUs to access the licensed bands assigned to the PUs while the PUs are inactive or the SUs can coexist with the PUs in a non-interfering manner [16,17,18,19]. The above operation paradigms for cognitive radio are known as opportunistic spectrum access and spectrum sharing [20,21]. In [22], the robust max-min fairness resource allocation in opportunistic spectrum access and sensing-based spectrum sharing is studied. For the easy implementation, the underlay paradigm is widely employed in spectrum sharing, and the precondition is that the SUs control the transmit power to avoid deterioration of the communication quality of PUs. Consequently, many authors focus on the efficient power allocation approaches for the satellite users in the uplink. In [23], the novel power and rate allocation approach for multi-users is proposed for the cognitive satellite uplink, where satellite users reuse the channels of fixed-service terrestrial microwave systems. However, this approach cannot be applied in fading channels. Considering the fading channel condition, optimal power control approaches are presented in [24,25]. An interference-based constraint to the transmit power control of the satellite users is introduced in [24], which ensures an acceptable threshold for the interference-to-signal of the terrestrial link. In [26], delay-limited capacity and outage capacity are proposed for the real-time application in cognitive satellite links. However, the aim of the above-mentioned research is to share the channels by taking a strictly power control approach and does not consider the higher capacity performance and wide coverage of satellite networks, which are the main characteristics of satellite communication systems.

According to the data presented in [27], many of the prized spectrum channels that have been assigned to the terrestrial users lie idle. In this context, the SUs can access the channel based on its own benefit to get higher capacities if the primary users are idle, which is just the idea of opportunistic spectrum access. However, the premise is that the SUs have to accurately identify the work state of the PUs in time. The authors in [28] make an overview of spectrum sensing approaches according to the different detection methods. The issue of opportunistic spectrum access for multiple SUs is addressed for hybrid satellite-terrestrial networks in [29]. The optimal spectrum access based on energy harvesting is investigated in [30], which designs the variable optimistic sensing intervals considering the PU traffic statistics. However, it is obvious that the SUs cannot access the channel if there is no idle spectrum, thus the approach is not ideal for real-time video transmission, such as living streaming or video conferencing.

In this article, firstly, we present an integrated wireless sensor and sensing-based cognitive satellite terrestrial network, where the satellite user acts as the sink node in the wireless sensor network for gathering and transmitting sensing data by the satellite network. Meanwhile, the satellite user is also the cognitive SU in the satellite terrestrial network. Subsequently, sensing-based power allocation scheme in the cognitive satellite terrestrial network is introduced for the perfect sensing scenario and the imperfect sensing scenario, which aims to make the cognitive satellite user access the channel seamlessly through intelligent schemes. We use the ergodic capacity of the satellite user to evaluate the performance of the scheme, and the average interference power constraints of the satellite user are introduced to constrain the interference to the terrestrial user. For the nonlinear concave optimization problem of the ergodic capacity, we use the Lagrange duality method [31] to decompose the problem into several parallel sub-problems. Herein, an iterative algorithm for searching the optimal transmission power of the satellite user and the sensing time is proposed. Finally, simulation results show that, comparing the traditional opportunistic spectrum access, the satellite user can get higher ergodic capacities under the presented method. The following sections of the paper are structured as follows: In Section 2, the integrated wireless sensor and sensing-based cognitive satellite terrestrial system model and link budgets are introduced. In Section 3, the sensing-based power allocation scheme is formulated for the perfect and imperfect sensing scenarios, the numerical simulation results are presented in Section 4, and Section 5 concludes the paper.

## 2. System Model

### 2.1. System Model

**Figure 1:**
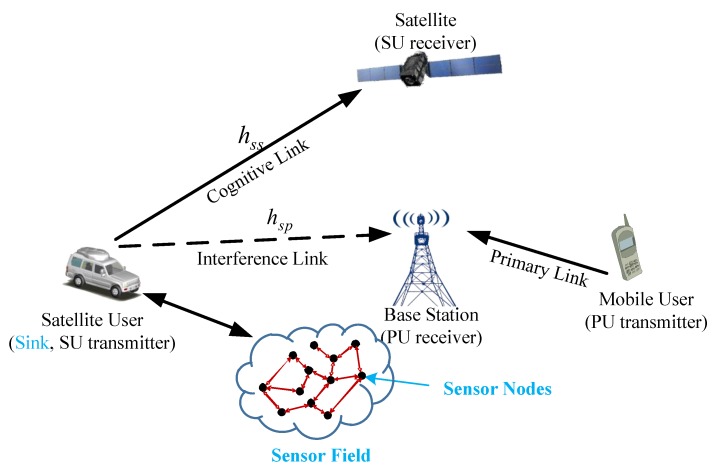
Integrated wireless sensor and cognitive satellite terrestrial network.

Figure 1 depicts the integrated wireless sensor and cognitive satellite terrestrial network architecture, which is composed of the cognitive satellite network, the terrestrial network, and the terrestrial sensor nodes. The satellite fixed/portable user acts as the sink of the wireless terrestrial sensor network, and they communicate with a number of wireless sensors via a radio link while providing the interface between the terrestrial sensor network and the satellite system. More details are presented as follows.

Terrestrial sensor nodes: In the sensor field, the sensor nodes monitor the terrestrial network state and transmit the monitoring results to the satellite user.

Terrestrial network: The terrestrial network plays the role of PU, and the link between the mobile user and base station, which is pre-allocated, is called the primary link.

Cognitive satellite network: The cognitive satellite network acts as the SU in the architecture. The satellite user, which acts as the sink of the wireless terrestrial sensor network, can dynamically access the cognitive link that was allocated to the terrestrial network according to the results of sensing results. In addition, $h_{ss}$ denotes the gain of the cognitive link. However, considering the impact of the antenna patterns and propagation issues, the satellite user would interfere the terrestrial network when the two systems working simultaneously and $h_{sp}$ refers to the gain of interference link.

Nowadays, WSN plays an important role in assisting us to understand the world, which is widely applied in environmental monitoring, health monitoring, and so on. In traditional WSNs, the sensor nodes collect and transmit the data to the fixed sink for the states in which the energy of the nodes closed to the fixed sink always deplete quickly, and the fixed sink is easy to be attacked. Some research pays attention to the mobile sink that just meets the requirements such as rescue mission, agricultural irrigation, and so on. Thus, the satellite user is introduced as a mobile sink in this paper.

$P_{t}$ and $P_{r}$ denote the transmit power of the satellite user and the satellite receive power, respectively. In addition, $P_{r}$ is formulated as:

(1) $$\begin{array}{c} \begin{array}{c} P_{r}=P_{t}G_{t}\left(\theta \right)G_{r}\left(\varphi \right)L_{ss}h_{ss}, \end{array} \end{array}$$

where $G_{t}\left(\theta \right)$ and $G_{r}\left(\varphi \right)$ represent the gain of the satellite user antenna and satellite receiver antenna, respectively. In addition, $G_{t}\left(\theta \right)$ and $G_{r}\left(\varphi \right)$ can be obtained as follows:

(2) $$\begin{array}{c} \begin{array}{c} G_{t}\left(\theta \right)=\left\{\begin{array}{c} G_{t,\max},\;\;\;0^{{}^{\circ}}<\theta <1^{{}^{\circ}},\\ 32-25\log\theta ,\;1^{{}^{\circ}}<\theta <48^{{}^{\circ}},\\ \;\;-10,\;\;\;48^{{}^{\circ}}<\theta <180^{{}^{\circ}}, \end{array}\right. \end{array} \end{array}$$

where θ is the elevation angle of the satellite user, $G_{t,\max}$ is the maximum beam gain at the onboard antenna boresight:

(3) $$\begin{array}{c} \begin{array}{c} G_{r}\left(\varphi \right)=G_{r,\max}{\left(\frac{J_{1}\left(u\right)}{2u}+36\frac{J_{3}\left(u\right)}{u^{3}}\right)}^{2}, \end{array} \end{array}$$

where φ is the angle between the beam center of the satellite antenna and connection from the satellite user to satellite, and J(·) is the Beseel function. In addition, $u=2.07123\frac{\sin\varphi }{\sin\varphi_{3\mathrm{dB}}}$, where $\varphi_{3\mathrm{dB}}$ denotes 3 dB angle. $L_{ss}$ represents the free space loss, which mainly relates to frequency and distance. Considering that the satellite communication is line-of-sight and the user operates in various propagation environments, we adopted the Shadowed-Riced fading channel model to express the channel characteristic. Therefore, according to [32], the probability density function(PDF) of $h_{ss}$ is formulated as:

(4) $$\begin{array}{c} \begin{array}{c} f_{h_{ss}}\left(x\right)=\alpha \exp\left(\beta x\right){}_{1}F_{1}\left(m_{ss},1,\lambda x\right) \end{array} \end{array}$$

where ${}_{1}F_{1}\left(\cdot ,\cdot ,\cdot \right)$ is the confluent hypergeometric function, and α, β and λ are formulated respectively as:

(5) $$\begin{array}{c} \begin{array}{c} \alpha =\frac{1}{2b_{ss}}{\left(\frac{2b_{ss}m_{ss}}{2b_{ss}m_{ss}+\Omega_{ss}}\right)}^{m_{ss}}\\ \beta =-\left(\frac{1}{2b_{ss}}\right)\\ \lambda =\frac{\Omega_{ss}}{2b{}_{ss}\left(2b_{ss}m_{ss}+\Omega_{ss}\right)}, \end{array} \end{array}$$

where $2b_{ss}$ is the average power of the scatter component, $m_{ss}$ denotes the Nakagami fading parameter, and $\Omega_{ss}$ is the average power of the line-of-sight (LOS) component. Similarly, the power of the interference link $P_{i}$ that is between the satellite user to the terrestrial base station can be calculated as:

(6) $$\begin{array}{c} \begin{array}{c} P_{i}=P_{t}G_{t}\left(\theta^{\prime }\right)G_{BS}L_{sp}h_{sp}, \end{array} \end{array}$$

where $G_{t}\left(\theta^{\prime }\right)$ is transmit antenna gain, the off-axis angle $\theta^{\prime }=\arccos\left(\cos\left(\theta \right)\cos\left(\psi \right)\right)$, and ψ represents the angle between the projected main lobe of the satellite user and the BS [33]. In addition, $G_{BS}$ is the gain of base station receiver antenna, and $L_{sp}$ is free space loss. In addition, the Nakagami fading model is adopted to present the interference link, and the PDF of $h_{sp}$ is as follows:

(7) $$\begin{array}{c} \begin{array}{c} f_{h_{sp}}\left(x\right)=\frac{{\left(\frac{m_{sp}}{\Omega_{sp}}\right)}^{m_{sp}}x^{m_{sp}-1}}{\Gamma \left(m{}_{sp}\right)}\exp\left(-\frac{m_{sp}}{\Omega_{sp}}x\right), \end{array} \end{array}$$

where $\Gamma \left(m{}_{sp}\right)$ is the Gamma function [20], $\Omega_{sp}$ is the signal average power, and $m_{sp}$ is the Nakagami fading parameter. Moreover, the Additive Gauss White Noise (AWGN) is considered as independent and zero mean, and its distribution is denoted as $CN\left(0,N_{0}\right)$.

Additionally, due to the large distance between the primary terrestrial user to the satellite and the limited power control of the terrestrial user, the interference caused by primary terrestrial user to the satellite can be neglected [20]. In addition, it is assumed that it is possible to obtain the perfect Channel State Information (CSI) of $h_{ss}$ and $h_{sp}$ by satellite user [34], which can be accomplished through the database or the training model [26].

### 2.2. Transmission Model

**Figure 2:**
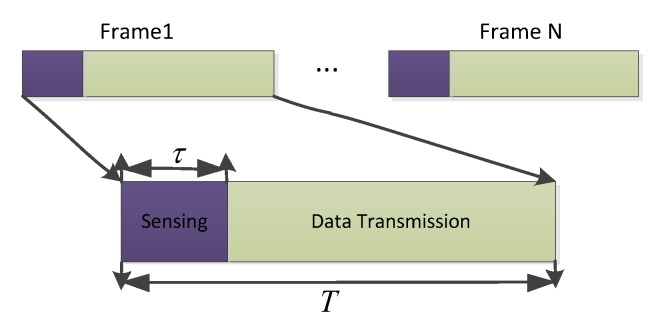
Periodic spectrum sensing frame structure.

In this paper, we design the periodic frame structure, and the time duration of each frame is *T*. As shown in Figure 2, each frame duration consists of two parts: the sensing slot and the data transmission slot. During the sensing slot τ, the satellite user that acts as the sink of the sensor network can monitor the terrestrial network state through the sensor data. In addition, during the transmission slot T−τ, the satellite user adapts its power control scheme to access the channel assigned to a terrestrial network without degrading the communication quality of the terrestrial user.

The probabilities of the busy/idle states of terrestrial network are denoted by $P(H_{b})$ and $P(H_{i})$, respectively, and obviously $P\left(H_{b}\right)+P\left(H_{i}\right)=1$. The paper introduces $H_{b}/H_{i}$ to represent the sensor results of busy/idle state of the terrestrial network, and the expressions are as follows:

(8) $$\begin{array}{c} \begin{array}{c} H_{b}:y\left(i\right)=cx\left(i\right)+n\left(i\right),\\ H_{i}:y\left(i\right)=n\left(i\right), \end{array} \end{array}$$

where i=1,2,⋯,N, *N* is the sample number of one frame and follows $N\geq \tau f_{s}$, $f_{s}$ is sample frequency, y(i) is the signal received by the satellite user that acts as the sensor sink, x(i) denotes the signal sent by the terrestrial user, *c* represents the channel gain of sensor network which is changed in different frame but fixed in one frame, and n(i) is AWGN with zero mean and variance $\sigma_{n}^{2}$.

Under this assumption, the sensing results contain two types, which are called busy state and idle state. The sensing result of busy state can be caused by two possibilities: (a) when the primary terrestrial network is busy, which happens with the probability $P(H_{b})$, and the sensing result is correct. The procedure is defined as the perfect detection and the probability of detection is denoted by $P_{d}$; (b) when the primary terrestrial network is idle, which happens with the probability $P(H_{i})$, but the sensing result is false. It is defined as a false alarm and the probability of the sensing result is represented by $P_{f}$. Likewise, the idle state also contains two possible causes: (c) if the primary terrestrial network is busy with the probability of $P(H_{b})$, but the sensor result is idle, the procedure is called error detection with probability of $\left(1-P_{d}\right)$; and (d) the sensing result is idle and the primary terrestrial network is just idle too, and the detection probability is denoted as $\left(1-P_{f}\right)$.

Considering the lowest complexity and the high efficiency [35], the energy detection approach is adopted to deal with the sensor data. In addition, then, the detection probability $P_{d}$ and the false detection probability $P_{f}$ can be calculated as [21]:

(9) $$\begin{array}{c} \begin{array}{c} P_{d}\left(\varepsilon ,\tau \right)=Q\left(\left(\frac{\varepsilon }{\sigma_{n}^{2}}-\gamma -1\right)\sqrt{\frac{\tau f_{s}}{2\gamma +1}}\right), \end{array} \end{array}$$

(10) $$\begin{array}{c} \begin{array}{c} P_{f}\left(\varepsilon ,\tau \right)=Q\left(\left(\frac{\varepsilon }{\sigma_{n}^{2}}-1\right)\sqrt{\tau f_{s}}\right), \end{array} \end{array}$$

where ε is the detection threshold, γ refers to the signal-to-noise ratio (SNR) of received sensing signals, and Q(·) denotes the complementary distribution function of the standard Gaussian, i.e.,

(11) $$\begin{array}{c} Q\left(x\right)=\frac{1}{\sqrt{2\pi }}\int_{x}^{\infty }\exp\left(-\frac{t^{2}}{2}\right)dt. \end{array}$$

The satellite user can adopt a different power control scheme to access the channel based on the sensing results. Precisely, the satellite user accesses the channel according to the following rules: if the sensing result shows that the primary terrestrial network is idle, the satellite user can enhance the power to access the channel without considering the interference in the primary terrestrial network; otherwise, if the state of primary terrestrial network is evaluated to be busy, the satellite user adopts its power to constrain the interference to the terrestrial network. $P_{s}^{\left(b\right)}$ and $P_{s}^{\left(i\right)}$ are used to represent the transmission power of the satellite user when the sensing result is busy/idle, respectively. It is obvious that $P_{s}^{\left(b\right)}<P_{s}^{\left(i\right)}$.

As mentioned above, the capacity of the satellite network can be described as follows: if terrestrial network is idle and the sensing result is just correct, the capacity of satellite network is calculated as:

(12) Cii=Blog21+Ps(i)Gt(θ)Gr(φ)LsshssPs(i)Gt(θ)Gr(φ)LsshssN0N0,

where *B* represents the bandwidth of the channel. Otherwise, if there is a false alarm, the capacity can be presented as:

(13) Cib=Blog21+Ps(b)Gt(θ)Gr(φ)LsshssPs(b)Gt(θ)Gr(φ)LsshssN0N0.

Meanwhile, in the perfect detection conditions, which means, if the terrestrial network is active and the sensing result is correct, the capacity of satellite network is calculated as follows:

(14) Cbb=Blog21+Ps(b)Gt(θ)Gr(φ)LsshssPs(b)Gt(θ)Gr(φ)LsshssN0N0.

However, if the sensing result is false, the capacity is represented as:

(15) Cbi=Blog21+Ps(i)tGt(θ)Gr(φ)LsshssPs(i)tGt(θ)Gr(φ)LsshssN0N0.

## 3. Problem Formulations

In this section, we present the proposed optimal scheme for the transmission power of the cognitive satellite user firstly. In addition, then, the perfect sensing and imperfect sensing conditions are considered, respectively. Ergodic capacity is adopted as the metric to measure the performance of the satellite network with average interference power constraints in the paper. As mentioned above, there are four possible states, and the ergodic capacity of cognitive satellite uplink can be calculated as follows:

(16) $$\begin{array}{c} C=E\left\{\frac{T-\tau }{T}\left[P\left(H_{i}\right)\left(1-P_{f}\right)C_{ii}+P\left(H_{i}\right)P_{f}C_{ib}+P\left(H_{b}\right)P_{d}C_{bb}+P\left(H_{b}\right)\left(1-P_{d}\right)C_{bi}\right]\right\} \end{array}$$

where E{·} denotes the expectation. The expression Equation (16) is the objective function. To regulate the transmit power limit of the satellite user, the average transmit power is adopted as follows:

(17) $$\begin{array}{c} E\left\{P_{s}^{\left(i\right)}\right\}P\left(H_{i}\right)\left(1-P_{f}\right)+E\left\{P_{s}^{\left(b\right)}\right\}P\left(H_{i}\right)P_{f}+E\left\{P_{s}^{\left(i\right)}\right\}P\left(H_{b}\right)\left(1-P_{d}\right)+E\left\{P_{s}^{\left(b\right)}\right\}P\left(H_{b}\right)P_{d}\leq P_{av} \end{array}$$

where $P_{av}$ denotes the average transmit power limit of the satellite user. Furthermore, the average interference power constraints of the satellite user are as follows:

(18) $$\begin{array}{c} E\left\{P_{s}^{\left(i\right)}G_{t}\left(\theta^{\prime }\right)G_{BS}L_{sp}h_{sp}\right\}\left(1-P_{d}\right)+E\left\{P_{s}^{\left(b\right)}G_{t}\left(\theta^{\prime }\right)G_{BS}L_{sp}h_{sp}\right\}P_{d}\leq I_{av}, \end{array}$$

where $I_{av}$ refers to the average interference power constraint limit.

Therefore, the sensing-based dynamic spectrum sharing method can be formulated as the following optimization problem:

(19) $$\begin{array}{c} \begin{array}{c} \max_{\left\{P_{s}^{\left(i\right)},P_{s}^{\left(b\right)},\tau \right\}}C,\\ s.t.\left(17\right)\;and\;\left(18\right). \end{array} \end{array}$$

The expression Equation (19) is not a linear convex optimization problem, which contains two complicated constraints. Then, $P_{d}$ and $P_{f}$ are the *Q* functions and taking over the sensing slot duration τ. In addition, *C* is related to two random variables $h_{ss}$ and $h_{sp}$. Therefore, we modify (19) under the perfect sensing condition and the imperfect sensing condition, respectively.

### 3.1. Sensing-Based Spectrum Sharing under Perfect Sensing Conditions

In perfect sensing conditions, where the satellite user can correctly detect the terrestrial network state without a false alarm in the variable short sensing time τ, and we have $P_{d}=1$, $P_{f}=0$ and τ=0. Thus, Equation (19) can be simplified to Equation (20),

(20) $$\begin{array}{c} \begin{array}{c} C=\max_{\left\{P_{s}^{\left(i\right)},P_{s}^{\left(b\right)}\right\}}E\left\{C_{ii}\right\}P\left(H_{i}\right)+E\left\{C_{bb}\right\}P\left(H_{b}\right),\\ s.t.\left\{\begin{array}{c} E\left\{P_{s}^{\left(i\right)}\right\}P\left(H_{i}\right)+E\left\{P_{s}^{\left(b\right)}\right\}P\left(H_{b}\right)\leq P_{av},\\ E\left\{P_{s}^{\left(b\right)}G_{t}\left(\theta^{\prime }\right)G_{BS}L_{sp}h_{sp}\right\}\leq I_{av}. \end{array}\right. \end{array} \end{array}$$

Obviously, the objective function and constraints are not related to the sensing time τ. Moreover, the objective function is a concave function with respect to the power $P_{s}^{\left(i\right)}$ and $P_{s}^{\left(b\right)}$. The Lagrangian duality method is adopted to solve Equation (20) since the duality gap is zero [31]. The Lagrangian function of Equation (20) can be formulated as

(21) $$\begin{array}{c} \begin{array}{c} L\left(P_{s}^{\left(i\right)},P_{s}^{\left(b\right)},\lambda ,\mu \right)=E\{C_{ii}\}P\left(H_{i}\right)+E\{C_{bb}\}P\left(H_{b}\right)-\lambda \{E\left\{P_{s}^{\left(i\right)}\right\}P\left(H_{i}\right)+E\left\{P_{s}^{\left(b\right)}\right\}P\left(H_{b}\right)-P_{av}\}\\ -\mu \{E\{P_{s}^{\left(b\right)}G_{t}\left(\theta^{\prime }\right)G_{BS}L_{sp}h_{sp}\}-I_{av}\}, \end{array} \end{array}$$

where λ and μ are the non-negative Lagrangian multipliers. In addition, the Lagrangian dual function is defined as:

(22) $$\begin{array}{c} g\left(\lambda ,\mu \right)=\max_{\left\{0\leq P_{s}^{\left(b\right)},0\leq P_{s}^{\left(i\right)}\right\}}L\left(P_{s}^{\left(i\right)},P_{s}^{\left(b\right)},\lambda ,\mu \right). \end{array}$$

The dual function Equation (22) serves as an upper bound on the optimal value of Equation (20). Then, the dual problem can be defined as:

(23) $$\begin{array}{c} \min_{\lambda ,\mu }g\left(\lambda ,\mu \right). \end{array}$$

Similar to [36], Equation (23) can be decomposed into multiple parallel subdual functions as follows:

(24) $$\begin{array}{c} Subproblem1\left(SP1\right):\max_{P_{s}^{\left(i\right)}\geq 0}E\{C_{ii}\}-\lambda E\left\{P_{s}^{\left(i\right)}\right\}, \end{array}$$

(25) $$\begin{array}{c} Subproblem2\left(SP2\right):\max_{P_{s}^{\left(b\right)}\geq 0}E\{C_{bb}\}-\lambda E\left\{P_{s}^{\left(b\right)}\right\}-\mu E\left\{P_{s}^{\left(b\right)}G_{t}\left(\theta^{\prime }\right)G_{BS}L_{sp}h_{sp}\right\}+\mu I_{av}. \end{array}$$

According to the Karush-Kuhn-Tucker (KKT) conditions, the optimal transmission powers for sensing-based opportunistic channel access scheme can be formulated as:

(26) $$\begin{array}{c} P_{s}^{\left(i\right)}={\left(\frac{N_{0}B}{\lambda \ln2}-\frac{N_{0}}{G_{t}\left(\theta \right)G_{r}\left(\varphi \right)L_{ss}h_{ss}}\right)}^{+}, \end{array}$$

(27) $$\begin{array}{c} P_{s}^{\left(b\right)}={\left(\frac{B}{\ln2\left[\lambda +\mu G_{t}\left(\theta^{\prime }\right)G_{BS}L_{sp}h_{sp}\right]}-\frac{N_{0}}{G_{t}\left(\theta \right)G_{r}\left(\varphi \right)L_{ss}h_{ss}}\right)}^{+}, \end{array}$$

where ${\left(x\right)}^{+}$ means the maximum between 0 and *x*. Suppose that $h_{ss}^{\prime }=G_{t}\left(\theta \right)G_{r}\left(\varphi \right)L_{ss}h_{ss}$ and $h_{sp}^{\prime }=G_{t}\left(\theta^{\prime }\right)G_{BS}L_{sp}h_{sp}$, and they represent the channel gain of cognitive link and interference link considering the impact of the antenna patterns and propagation issues, respectively. It is obvious that transmitter power is proportional to the *B* and $h_{ss}^{\prime }$ for all states. Conversely, if the terrestrial network is monitored to be busy, the cognitive satellite user obtains higher transmission power when $h_{sp}^{\prime }$ is lower, since the more severe fading of the interference link is, the less influence of the satellite users on the terrestrial network.

Now, we can evaluate Equation (23) via the solutions of Equations (26) and (27) for the fixed λ and μ, and minimize the dual function $g\left(\lambda ,\mu \right)$ by updating λ and μ through Algorithm 1, which uses the subgradient method at each iteration.

**Algorithm 1** Iterative Power Allocation Algorithm under Perfect Sensing Conditions.

**Set parameters:**
$\sigma_{\lambda }>0,\sigma_{\mu }>0$: Error tolerances;α>0, β>0: Step sizes.*m*: Iteration number;
**Initialization:**
$\varepsilon_{\lambda }=\lambda_{0}$, $\varepsilon_{\mu }=\mu_{0}$, m=1;**While**$\varepsilon_{\lambda }>\sigma {}_{\lambda }$ and $\varepsilon_{\mu }>\sigma_{\mu }$    Calculate $P_{s,m}^{\left(i\right)}$ and $P_{s,m}^{\left(b\right)}$ using (26) and (27), respectively;    Update λ and μ by subgradient method as follows:    $\lambda_{m+1}=\max\left(0,\lambda_{m}-\alpha \left(P_{av}-E\left\{P_{s,m}^{\left(i\right)}\right\}P\left(H_{i}\right)-E\left\{P_{s,m}^{\left(b\right)}\right\}P\left(H_{b}\right)\right)\right)$    $\mu_{m+1}=\max\left(0,\mu_{m}-\beta \left(I_{av}-E\left\{G_{t}\left(\theta^{\prime }\right)G_{BS}L_{sp}h_{sp}P_{s,m}^{\left(b\right)}\right\}\right)\right)$;    $\varepsilon_{\lambda }=abs\left(\lambda_{m+1}-\lambda_{m}\right)$;    $\varepsilon_{\mu }=abs\left(\mu_{m+1}-\mu_{m}\right)$;**End**;$P_{s}^{\left(i\right)*}=P_{s,m}^{\left(i\right)},P_{s}^{\left(b\right)*}=P_{s,m}^{\left(b\right)}$;$\lambda^{*}=\lambda_{m+1};\mu^{*}=\mu_{m+1}$.


### 3.2. Sensing-Based Spectrum Sharing under Imperfect Sensing Conditions

In Section 3.1, the perfect sensing scenario is presented, and the optimal capacity is the upper bound of Equation (19). Herein, the imperfect sensing condition, where the sensing time τ is not zero, is considered. To protect the primary user in a conventional sensing-based spectrum cognitive radio model, the detection probability $P_{d}$ is usually larger than detection threshold ${\bar{P}}_{d}$, and ${\bar{P}}_{d}$ is usually close to 1 but less than 1. For instance, in the IEEE 802.22 WRAN, ${\bar{P}}_{d}$ is 0.9 as the SNR is –20 dB [37]; therefore, $P_{d}$ is usually chosen to be larger than 0.9, and $P_{f}$ is controlled to be very small. Under the assumptions, we can introduce the fact that $P_{d}$ is larger than ${\bar{P}}_{d}$, close to 1, and $P_{f}$ is closed to zero. Therefore, Equation (19) can be simplified as follows:

(28) $$\begin{array}{c} \begin{array}{c} C=\max_{\left\{P_{s}^{\left(i\right)},P_{s}^{\left(b\right)},\tau \right\}}E\left\{\frac{T-\tau }{T}\left[P\left(H_{i}\right)\left(1-P_{f}\right)C_{ii}+P\left(H_{b}\right)P_{d}C_{bb}\right]\right\},\;\left(e0\right)\\ s.t.\left\{\begin{array}{c} E\left\{P_{s}^{\left(i\right)}\right\}P\left(H_{i}\right)\left(1-P_{f}\right)+E\left\{P_{s}^{\left(b\right)}\right\}P\left(H_{b}\right)P_{d}\leq P_{av},\;\;\left(e1\right)\\ E\left\{P_{s}^{\left(b\right)}G_{t}\left(\theta^{\prime }\right)G_{BS}L_{sp}h_{sp}\right\}P_{d}\leq I_{av},\;\;\;\;\;\;\left(e2\right)\\ 0\leq \tau \leq T.\;\;\;\;\;\;\;\;\;\;\;\;(e3) \end{array}\right. \end{array} \end{array}$$

Obviously, (e0) is a convex optimization problem with respect to $P_{s}^{\left(i\right)}$ and $P_{s}^{\left(b\right)}$. In the following, we prove that it is concave in τ, when τ belongs to the area of $\left(0,T\right)$.

**Proposition** **1.** *When $\tau \in \left(0,T\right)$, $P_{f}$ is decreasing and convex in τ, and $P_{d}$ is increasing and concave in τ, respectively.*

**Proof of Proposition 1.**  From Equation (9) and Equation (10), when the target detection probability is ${\bar{P}}_{d}$, $P_{f}$ can be determined by $P_{f}\left(\tau \right)=Q\left(\sqrt{2\gamma +1}Q^{-1}\left({\bar{P}}_{d}\right)+\sqrt{\tau f_{s}}\gamma \right)$, and notice $\beta =\sqrt{2\gamma +1}Q^{-1}\left({\bar{P}}_{d}\right)$. We have

(29) $$\begin{array}{c} P_{f}\left(\tau \right)=Q\left(\beta +\sqrt{\tau f_{s}}\gamma \right). \end{array}$$

Differentiating $P_{f}\left(\tau \right)$ with respect to τ gives:

(30) $$\begin{array}{c} P_{f}^{\prime }\left(\tau \right)=\frac{dP_{f}\left(\tau \right)}{d\tau }=-\frac{\gamma \sqrt{f_{s}}}{2\sqrt{2\pi \tau }}\exp\left(-\frac{{\left(\beta +\sqrt{\tau f_{s}}\gamma \right)}^{2}}{2}\right). \end{array}$$

For τ>0, $P_{f}^{\prime }\left(\tau \right)<0$ and $P_{f}\left(\tau \right)$ are decreasing. In addition, when $P_{f}\left(\tau \right)\leq 0.5$, we have $\beta +\sqrt{\tau f_{s}}\gamma \geq 0$. Together with Equation (30), $P_{f}^{\prime }\left(\tau \right)$ is monotonically increasing in τ when $P_{f}\left(\tau \right)\leq 0.5$. Therefore, $P_{f}\left(\tau \right)$ is decreasing and convex in $\tau \in \left(0,T\right)$ when $P_{f}\left(\tau \right)\leq 0.5$. Similarly, for the range of τ, when $P_{d}\left(\tau \right)\geq 0.5$, $P_{d}\left(\tau \right)$ is increasing and concave. □

**Proposition** **2.** *When $\tau \in \left(0,T\right)$, $P_{f}\left(\tau \right)$ is decreasing and convex and $P_{d}\left(\tau \right)$ is increasing and concave, (e0) is also concave.*

**Proof of Propostion 2.**  Denote

(31) $$\begin{array}{c} R\left(\tau \right)=\frac{T-\tau }{T}\left[P\left(H_{i}\right)\left(1-P_{f}\right)C_{ii}+P\left(H_{b}\right)P_{d}C_{bb}\right]. \end{array}$$

Differentiating R(τ) with respect to τ gives:

$$\begin{array}{c} R^{\prime }\left(\tau \right)=-\frac{1}{T}\left[P\left(H_{i}\right)\left(1-P_{f}\left(\tau \right)\right)C_{ii}+P\left(H_{b}\right)P_{d}\left(\tau \right)C_{bb}\right]\\ +\frac{T-\tau }{T}\left[P\left(H_{i}\right)\left(-{P^{\prime }}_{f}\left(\tau \right)\right)C_{ii}+P\left(H_{b}\right){P^{\prime }}_{d}\left(\tau \right)C_{bb}\right]\\ =P\left(H_{i}\right)C_{ii}\left[-\frac{1}{T}+\frac{P_{f}\left(\tau \right)}{T}-\left(1-\frac{\tau }{T}\right)P_{f}^{\prime }\left(\tau \right)\right]-P\left(H_{b}\right)C_{bb}\left[\frac{P_{d}\left(\tau \right)}{T}-\left(1-\frac{\tau }{T}\right)P_{d}^{\prime }\left(\tau \right)\right]. \end{array}$$

Proposition 1 shows that, when $\tau \in \left(0,T\right)$, if $P_{f}\left(\tau \right)\leq 0.5$, $P_{f}\left(\tau \right)$ is decreasing, $P_{f}^{\prime }\left(\tau \right)<0$ and $P_{f}^{\prime }\left(\tau \right)$ is monotonically increasing; similarly, if $P_{d}\left(\tau \right)\geq 0.5$, $P_{d}\left(\tau \right)$ is increasing, $P_{d}^{\prime }\left(\tau \right)>0$ and $P_{d}^{\prime }\left(\tau \right)$ is monotonically decreasing. Therefore, $R^{\prime }\left(\tau \right)$ is decreasing when τ∈(0,T), which further indicates that R(τ) is concave in τ. Since the concavity is not affected by the expectation, (e0) is a concavity function with respect to τ. □

It can be verified that the constraints Equations (e1) and (e2) are concave with τ by the same method. Therefore, Equation (28) is a convex optimization problem with $P_{s}^{\left(i\right)}$, $P_{s}^{\left(b\right)}$, and τ. Without loss of generality, $P(H_{b})$ is supposed to be very small, since it is valuable to explore the spectrum sharing approach. Consequently, the function *C* can be approximated as:

(32) $$\begin{array}{c} \tilde{C}=\max_{\tau }E\left\{\frac{T-\tau }{T}\left[P\left(H_{i}\right)\left(1-P_{f}\right)C_{ii}\right]\right\}. \end{array}$$

According to Equations (9) and (10), $P_{d}$ and $P_{f}$ are related to the sensing time τ and the detect threshold ε. For a given τ, there is a $\varepsilon_{0}$ such that $P_{d}\left(\varepsilon_{0},\tau \right)={\bar{P}}_{d}$. If we choose $\varepsilon_{1}<\varepsilon_{0}$, thus $P_{d}\left(\varepsilon_{1},\tau \right)>{\bar{P}}_{d}$ and $P_{f}\left(\varepsilon_{1},\tau \right)>P_{f}\left(\varepsilon_{0},\tau \right)$. Then, we have $\tilde{C}\left(\varepsilon_{1},\tau \right)<\tilde{C}\left(\varepsilon_{0},\tau \right)$. Therefore, the maximum solution of Equation (28) can be calculated when $P_{d}\left(\varepsilon_{0},\tau \right)={\bar{P}}_{d}$. Similarly, Equation (28) can be solved by the modified Algorithm 1 as follows: firstly, we initialize τ, λ, μ, and calculate $P_{d}\left(\tau \right)$, $P_{f}\left(\tau \right)$. Subsequently, the values of λ, μ can be updated by the subgradient algorithm. Finally, we calculate $P_{s}^{\left(i\right)}$, $P_{s}^{\left(b\right)}$, and *C* under the τ and update the new τ. These steps are repeated until all the τ is calculated.

## 4. Simulations and Discussion

In this section, we evaluate the performance of the proposed schemes under the two conditions. The simulation parameters are shown in Table 1 [8,21,22,23,32]. Moreover, all the simulation results are obtained through Monte Carlo simulations.

### 4.1. Perfect Sensing Conditions

**Figure 3:**
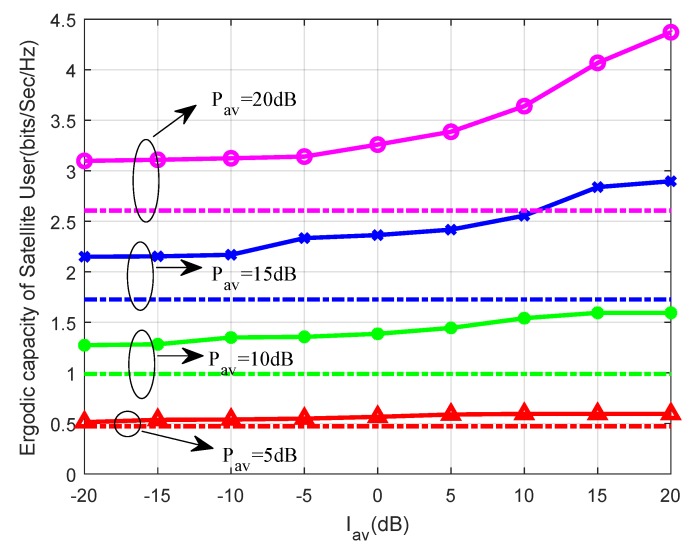
Ergodic capacity versus $I_{av}$ for different $P_{av}$ with $m_{sp}=1$ and $P\left(H_{i}\right)=0.6$.

In this part, the continuous lines and the dash dot lines refer to the capacities of the proposed method and the opportunistic spectrum access approach, respectively. Figure 3 shows the ergodic capacities of the paper proposed increasing with $P_{av}$ and $I_{av}$. However, the capacities of opportunistic spectrum access approach only increase with $P_{av}$. This is because the satellite user can only access the channel with the fixed transmission power while the terrestrial user is idle without considering the power constraint $I_{av}$ in the opportunistic spectrum access approach. In addition, it is obvious that the proposed approach leads to higher values of ergodic capacities compared with the opportunistic spectrum access approach in all the considered scenarios.

**Figure 4:**
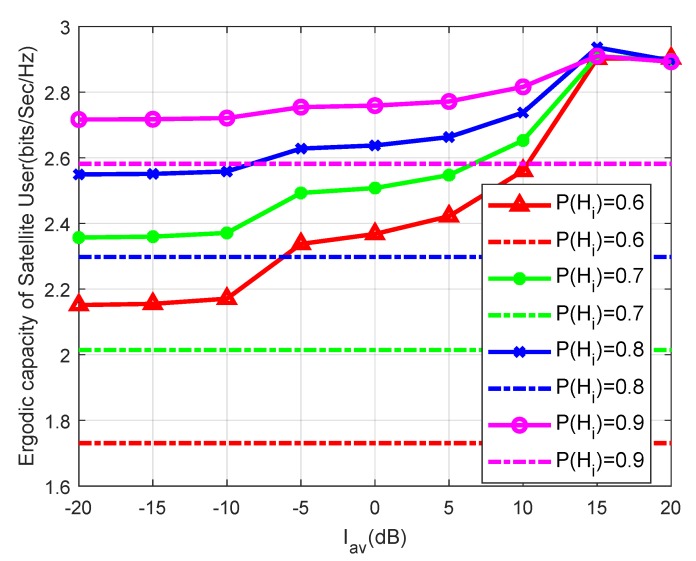
Ergodic capacity versus $I_{av}$ for different $P(H_{i})$ with $P_{av}=15\;\mathrm{dB}$ and $m_{sp}=1.$

In Figure 4, it can be observed that the satellite network capacities increase with the increase of $P\left(H_{i}\right)$. This is due to the fact that the more idle the terrestrial user is, the larger the ergodic capacity that the satellite user can obtain. Furthermore, under the same $P(H_{i})$, the ergodic capacities of the proposed approach are significantly higher than that of the opportunistic spectrum access approach. However, the capacity gap between the two approaches is smaller when the $P(H_{i})$ is higher; this is because the opportunistic spectrum access approach has excellent performance if the terrestrial user is always idle. Moreover, we can see that the ergodic capacities of the proposed method become saturated when the $I_{av}$ is large, since the transmit power $P_{av}$ becomes the main constraint.

**Figure 5:**
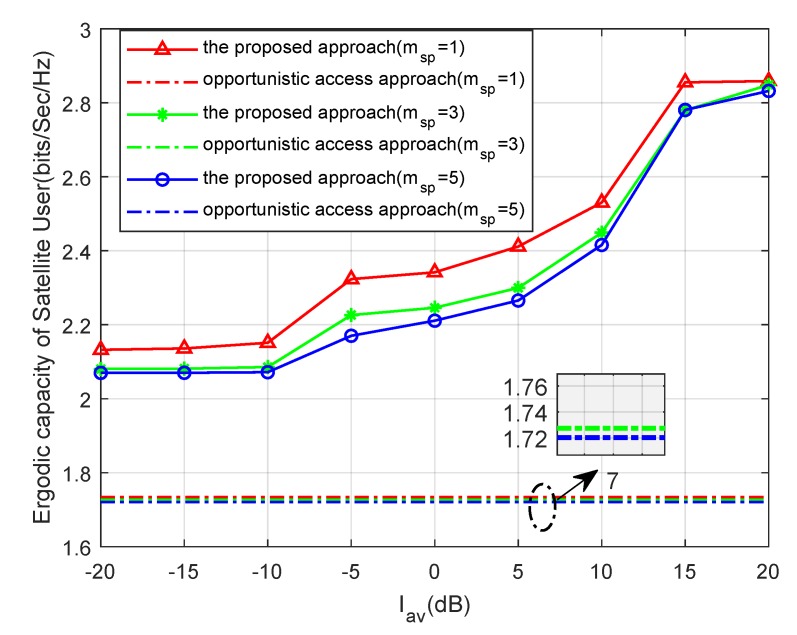
Ergodic capacity versus $I_{av}$ for different $m_{sp}.$

In Figure 5, we study the performance of the ergodic capacity of the satellite user versus $I_{av}$ for different $m_{sp}$ with $P\left(H_{i}\right)=0.6$ and $P_{av}=\;15\;\mathrm{dB}$. It is seen that ergodic capacity trends are consistent with the findings in Figure 4. Since the smaller values of $m_{sp}$ correspond to the more severe fading condition of the interference link, which is a benefit for the satellite user link, the ergodic capacity is inversely proportional to $m_{sp}$. The result is consistent with the results obtained in Equations (7) and (27).

### 4.2. Imperfect Sensing Conditions

**Figure 6:**
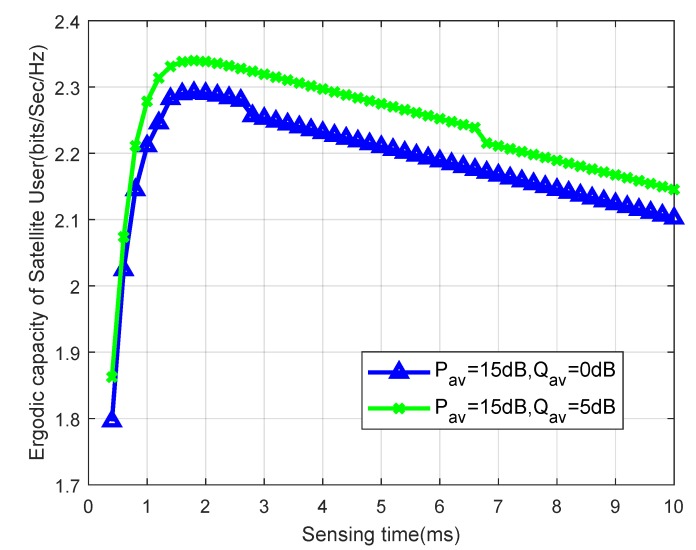
Ergodic capacity versus sensing time for different $I_{av}$ with $m_{sp}=1.$

Figure 6 depicts the ergodic capacities of cognitive satellite network versus the sensing time for different $I_{av}$ in imperfect conditions. It should be noted that the ergodic capacities are the concave function of the sensing time. Along with the sensing time increasing, the ergodic capacities increase at the beginning and decrease after the optimal sensing time, since the longer the sensing time, the smaller the transmission time.

**Figure 7:**
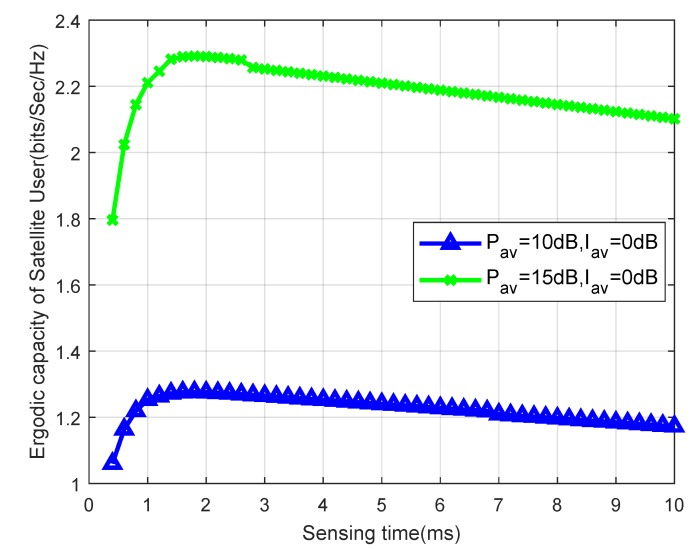
Ergodic capacity versus sensing time for different $P_{av}$ with $m_{sp}=1.$

The ergodic capacity of cognitive satellite network versus τ with different $P_{av}$ is illustrated in Figure 7. The trend of ergodic capacities is similar to Figure 6. However, the ergodic capacity gain in Figure 7 is larger than that in Figure 6. It is because $P_{av}$ has more impact on the ergodic capacity than $I_{av}$.

**Figure 8:**
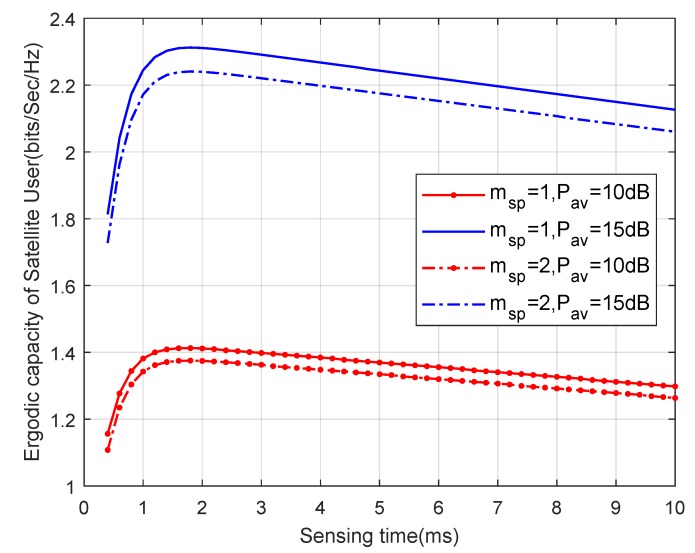
Ergodic capacity versus sensing time for different $P_{av}$ and $m_{sp}$ with $I_{av}=0$ dB.

In Figure 8, we present the ergodic capacity of the satellite user versus sensing time τ with different $P_{av}$ and $m_{sp}$. It can be clearly observed that the trend of ergodic capacities is consistent with the findings in Figure 6 and Figure 7, and the achievable ergodic capacity being higher corresponds to a larger $P_{av}$. Furthermore, it is noted that, with the difference of $m_{sp}$, the optimal sensing durations are almost the same. However, along with the decrease of $m_{sp}$, the ergodic capacity of the satellite user increases for the same $P_{av}$, since the smaller the value of $m_{sp}$, the more severe the fading conditions of the interference link become.

## 5. Conclusions

In this paper, we propose a novel satellite-based WSN, which integrates the WSN with the cognitive satellite terrestrial network. Then, the sensing-based spectrum sharing scheme in the cognitive satellite terrestrial network is presented for the perfect sensing condition and the imperfect sensing condition, respectively. For both scenarios, the ergodic capacity of the satellite uplink under the transmit and the interference power constraints is studied. In the context, the expression of the ergodic capacity can be formulated as a nonlinear fraction programming problem in both conditions, and the Lagrange duality method is adopted to solve the problem. Computer simulations have shown that, in a perfect sensing scenario, the proposed method can increase the channel capacity more than 20% when $P_{av}=10\;\mathrm{dB}$ compared with the traditional opportunistic spectrum access. In an imperfect sensing scenario, $P_{av}=15$ dB and $Q_{av}=5$ dB, the optimal sensing time achieving the highest ergodic capacity is about 2.34 ms when the frame duration is 10 ms. In future works, we will investigate the bounded CSI error model affect on the proposed method.

## Figures and Tables

**Table 1:** Simulation parameters.

Parameters	Values
signal frequency (*f*)	2 GHz
$G_{t,\max}^{}$	42.1 dB
$G_{r,\max}^{}$	52.1 dB
θ	$20^{{}^{\circ}}$
ψ	$50^{{}^{\circ}}$
$N_{0}$	0.01 W
satellite link distance ($d_{s}$)	35,786 km
interference link distance ($d_{I}$)	10 km
$b_{ss}$	0.126
$\Omega_{ss}$	0.835
$m_{ss}$	10.1
$\Omega_{sp}$	1
${\bar{P}}_{d}$	0.95
*T*	100 ms
